# Curative Effects of ZHENG-Based Fuzheng-Huayu Tablet on Hepatitis B Caused Cirrhosis Related to CYP1A2 Genetic Polymorphism

**DOI:** 10.1155/2013/302131

**Published:** 2013-10-24

**Authors:** Qing-Ya Li, Zhi-Zhong Guo, Xin Deng, Lie-Ming Xu, Yue-Qiu Gao, Wei Zhang, Xiao-Su Wang, Dong-Ying Xue, Yi-Yu Lu, Ping Liu, Shi-Bing Su

**Affiliations:** ^1^Research Center for TCM Complexity System, Shanghai University of TCM, Shanghai 201203, China; ^2^Key Laboratory of Viral Disease Prevention and Treatment of Traditional Chinese Medicine of Henan Province, Zhengzhou 450008, China; ^3^Henan University of TCM, Zhengzhou 450008, China; ^4^Ruikang Hospital of Guangxi University of TCM, Nanning, Guangxi 530011, China; ^5^Shanghai Shuguang Hospital, Shanghai University of TCM, Shanghai 200021, China; ^6^Shanghai Longhua Hospital, Shanghai University of TCM, Shanghai 201203, China; ^7^Shanghai Yueyang Hospital, Shanghai University of TCM, Shanghai 200437, China; ^8^Shanghai Putuo Hospital, Shanghai University of TCM, Shanghai 200060, China; ^9^Shanghai University of TCM, Shanghai 201203, China

## Abstract

*Aim*. To investigate the correlation of Fuzheng-Huayu tablet (FZHY) efficacy on chronic hepatitis B caused cirrhosis (HBC) and single nucleotide polymorphisms (SNPs) of CYP1A2. *Methods*. After 111 cases of HBC with 69 excess, 21 deficiency-excess, and 21 deficiency ZHENGs (ZHENG, also called traditional Chinese medicine syndrome) were treated by FZHY for 6 months, clinical symptoms, Child-Pugh score, and ZHENG score were observed. Three of the SNPs in CYP1A2 gene were detected and analyzed using SNaPshot assay. *Results*. In ZHENG efficacy between effective and invalid groups, there was significant difference (*P* < 0.001). The ZHENG deficiency was significantly correlated with FZHY efficacy (*P* < 0.05). AA genotype of CYP1A2-G2964A was significantly different with GG genotype (*P* < 0.05) between CYP1A2 Genotypes and FZHY efficacy on ZHENG. More importantly, GA plus AA genotype of CYP1A2-G2964A was significantly different with deficiency ZHENG (*P* < 0.05) between CYP1A2 genotypes and FZHY efficacy on ZHENG. *Conclusion*. FZHY improved ZHENG score of HBC, and these efficacies may relate to CYP1A2-G2964A sites. It was suggested that CYP1A2-G2964A locus is probably a risk factor for ZHENG-based FZHY efficacy in HBC.

## 1. Introduction

Hepatitis B virus (HBV) infection is a major health problem in China. It is one of the important reasons for virus-related liver diseases, such as chronic hepatitis B (CHB), liver cirrhosis (LC), and hepatocellular carcinoma [1]. Worldwide, there are 350 million HBV-infected people who have 15–25% risk of dying from the HBV-caused LC or HCC [2]. Five-year survival rate of patients with severe CHB and its caused cirrhosis is about 50% [3]. Clinically, it lacks the effective drugs for the therapy of hepatitis B which caused cirrhosis (HBC) so far. 

ZHENG, also known as traditional Chinese medicine (TCM) syndrome or TCM pattern, is a characteristic profile in clinical manifestations. Clinical treatments rely on the successful differentiation of ZHENG [4]. It has been reported that Fuzheng-Huayu tablet (FZHY), a Chinese herbal medicine formula, affected liver fibrosis [5–7]. Moreover, it was able to halt the progress of liver fibrosis through inhibiting the activation of hepatic satellite cells in animal model [8]. Recent study has found that FZHY decreases the levels of HA and ZHENG scores and improves the life quality of HBC patients and the ZHENG differentiation is related to the FZHY treatment [9]. However, the mechanism of FZHY efficacy on HBC is still unclear.

The cytochrome P450 (CYP450) is a vast superfamily of haem-containing monooxygenases. The members of this ubiquitous superfamily play an important role in the metabolism and biosynthesis of a wide range of exogenous drug compounds [10]. Because variations of single nucleotide polymorphisms (SNPs) in human CYP450 genes cause different drug effects and even adverse effects, studies on SNPs of human CYP450 genes can be used for indicating the most possible genes associated with human diseases and relevant therapeutic targets, predicting the drug efficacy and adverse drug response, investigating individual gene specific properties, and then providing personalized and optimal clinic therapies [11]. CYP1A2 is an important member of the cytochrome P450 superfamily [12], and it has been reported that Chinese herbal formula is associated with CYP1A2 genotype [13].

In this study, the aim was to evaluate FZHY efficacy on HBC through the outcome assessment of clinical symptoms, ALT, AST, Child-Pugh score, and ZHENG score and investigate the relationship between the CYP1A2 SNPs and FZHY efficacy on HBC.

## 2. Materials and Methods

### 2.1. Study Design

The study was designed to be a multicenter study, as a clinical outcome assessment, and was carried out to evaluate the efficacy of FZHY, reported in accordance to the 2010 CORSORT statements. The patients were conducted at 6 centers including Longhua, Shuguang, Yueyang, and Putuo hospitals in Shanghai and Ruikang hospital and Guangxi hospital of TCM in Guangxi in China. One-hundred and eleven of HBC patients were given FZHY. It was evaluated to the FZHY efficacy between past and pre treatments through the outcome assessment of clinical symptoms, ALT, AST, Child-Pugh score, and ZHENG score. Moreover, the correlation of CYP1A2 SNPs and FZHY efficacy on HBC between effective and invalid groups was analyzed. The invalid cases were as a control compared with the effective group in FZHY treatment. The study was conducted according to the guidelines of the Declaration of Helsinki and the principles of Good Clinical Practice (China), and we obtained the approval of Medical Ethics Committee in Shanghai Shuguang Hospital. The clinical trial registration number was NCT00543426, which could be found in the website of clinicaltrials.gov.

### 2.2. Patients and TCM Diagnosis

To carry out the study with 127 participants in FZHY group, all patients signed the informed consent before treatment. In the process of followup, 16 patients in FZHY group were lost; thus, data from the other 111 patients in FZHY group were available for analysis.

All of the 111 patients were Chinese yellow race. Their ages were from 18 to 65 years (mean ± SD: 49.51 ± 10.05). There were 79 male cases (71.17%) and 32 female cases (28.83%). The clinical information of HBC patients such as symptoms and signs was collected from the above 6 hospitals, and then ZHENGs were classified into 69 excess, 21 deficiency-excess, and 21 deficiency ZHENGs (Table 1), according to define of diagnosis, and ZHENG differentiation of liver cirrhosis [14]. In order to ensure the repeatability and reliability of ZHENG and symptom diagnoses, all patients were diagnosed by 3 senior TCM physicians separately in the same condition, and the final diagnosis was made by a TCM botanic physician. It was brung into the further study when the diagnoses were consistent.

### 2.3. Interventions

FZHY (SFDA approval no: Z20050546) were prepared and provided by Shanghai Sundise Medicine Technology Development Co., Ltd. (Shanghai, China). There was the same appearance and smell and 0.4 g per tablet in FZHY. The quality control and preparing standardization of FZHY were established and enforced according to previous report [15]. 

127 patients were conducted at 6 centers. One-hundred and eleven of HBC patients were given FZHY, every day 3 times oral, every time 1.6 g, for a total of takes of 6 months. The FZHY efficacy was evaluated between before and after treatments through the outcome assessment of clinical symptoms, ALT, AST, Child-Pugh score, and ZHENG score. 

### 2.4. Efficacy Evaluation

The HBC patients were treated by FZHY for 6 months. The Child-Pugh scores of the HBC were recorded and calculated by rating the following 5 parameters including serum levels of bilirubin and albumin, prothrombin time, ascites and encephalopathy, and divided into class A (5-6 points), B (7–9 points) and C (10–15 points) [16, 17]. The efficacy evaluation of FZHY on HBC was as effective: decrease or no change of Child-Pugh score; invalid: increase of Child-Pugh score. 

The efficacy evaluation of ZHENG was according to “Guideline for Clinical New Drug Research in Chinese Herbal Medicine” [18]. The standard of ZHENG outcome was as follows: ZHENG score as without, 0; light, 1; heavy, 2 points. The calculation formula: the efficacy index of ZHENG (*N*) = (before treatment score − after treatment score)/before treatment score × 100%. The efficacy evaluation standard of TCM syndrome: clinical cure: *N* ≥ 90%; excellent: *N* < 90%~>60%; effective: *N* ≤ 60%~>30%; invalid: *N* ≤ 30%. In the this study, the effective was *N* > 30% including above clinical cure, excellent, and effective.

### 2.5. Samples and DNA Extraction

Blood samples were obtained from all subjects with informed consent and ethical review board approval in accordance with the tenets of the Declaration of Helsinki. Each subject donated 1 mL peripheral blood samples, which were collected in K_2_EDTA tubes, and then the genomic DNA was isolated from each sample, using the TIANamp Blood DNA Kit (Tiangen Biotech, Beijing, China). Subsequently, the DNA was stored at −80°C for genotype analysis.

### 2.6. SNaPshot Assay

SNPs were genotyped using the ABI PRISM SNaPshot Multiplex Kit (ABI Co., Ltd, CA, USA) and ABI 3730 XL DNA Analyzer. All the analyses were performed as described previously [19]. The primer sequences (Table 2) were selected for amplification. SNaPshot analysis was conducted using an ABI PRISM SNaPshot Multiplex Kit (ABI). Amplification reactions were performed in a thermal cycler for 45 cycles of 20 s denaturing at 96°C, 5 s annealing at 50°C, and 30 s extension at 60°C. The amplified products were denatured at 95°C for 5 minutes and then separated by an ABI PRISM 3730 XL Genetic Analyzer. This analysis was performed using GeneMapper 4.0 Software.

### 2.7. Statistical Analysis

All clinical data were expressed as the mean ± SD. The Wilcoxon test was used for the evaluation of the FZHY efficacy on clinical symptom, ALT, AST, Child-Pugh score, and ZHENG sore in HBC. The correlation between genotypes and phenotypes was compared by the *χ* ^2^ test. *P* < 0.05 was considered statistically significant in all tests. 

## 3. Results

### 3.1. Efficacy Evaluation of FZHY

As shown in Table 3, the curative effect of FZHY on ALT, AST, Child-Pugh score, and ZHENG sore was detected in 111 HBC patients. The ALT, AST, Child-Pugh score, and ZHENG score were evaluated before and after drug therapy. There was significant difference (*P* < 0.001) between before and after FZHY treatment in ZHENG score. However, there it was not statistically significant (*P* > 0.05) between before and after FZHY treatment in ALT, AST, and Child-Pugh score. 

As shown in Table 4, the correlation was analyzed between ZHENG and FZHY efficacy on 111 HBC patients. There were 67 effective cases and 44 invalid cases, with the total effective rate of 60.36%. Among them, there were 18 effective cases and 3 invalid cases, with the total effective rate of 85.71% in deficiency ZHENG, and there was statistically significance (*P* < 0.05), and OR was 3.940 (95% CI: 1.095, 14.173) compared to excess or deficiency-excess syndromes.

### 3.2. Correlation between CYP1A2 Genotypes and FZHY Efficacy on Child-Pugh Scores in HBC

After SNaPshot assay was carried out, CYP1A2-T5347C, CYP1A2-G2964A, and CYP1A2-C733A were analyzed to clarify the correlation between CYP1A2 SNPs and FZHY effect. As shown in Table 5, the Child-Pugh score was analyzed between before and after treatment groups, and there were 25 effective cases and 86 invalid cases, with the total effective rate of 22.52%. Moreover, in the Child-Pugh scores between effective and invalid groups, the frequency of CC, CT, and TT genotypes at CYP1A2-T5347C locus was 70.80% versus 75.00%, 25.00% versus 23.80%, and 4.20% versus 1.20%; the frequency of GG, GA, and AA genotypes at CYP1A2-G2964A locus was 41.70% versus 53.50%, 45.80% versus 34.90%, and 12.50% versus 11.60%; the frequency of CC, CA, and AA genotypes at CYP1A2-C733A locus was 12.50% versus 10.50%, 37.50% versus 44.20%, and 50.00% versus 45.30%, respectively. However, there was no statistical significance (*P* > 0.05) between the two groups in the comparison of any genetic locus.

### 3.3. Correlation between CYP1A2 Genotypes and FZHY Efficacy on ZHENG Scores in HBC

As shown in Table 6, the ZHENG score was analyzed between before and after treatment groups, and there were 67 effective cases and 44 invalid cases, with the total effective rate of 60.36%. Moreover, in the efficacy index of ZHENG between effective and invalid groups, the frequency of GG, GA, and AA genotypes at CYP1A2-G2964A locus was 49.30% versus 52.30%, 44.80% versus 25.00%, and 4.50% versus 22.70%, respectively. There was significant difference (*P* < 0.05) in CYP1A2-G2964A locus at AA compared with GG genotype. OR was 4.783 (95% CI: 1.184, 19.321). The frequency of CC, CT, TT genotypes at CYP1A2-C5347T locus were 71.60% versus 72.70%, 25.40% versus 20.50% and 0 versus 4.50%, respectively. The frequency of CC, CA, AA genotypes at CYP1A2-C733A locus were 13.40% versus 6.80%, 40.30% versus 45.50% and 44.80% versus 47.70%, respectively. However, there were no statistically significant (*P* > 0.05) between the genotypes at CYP1A2-C5347T and -C733A sites and efficacy index of ZHENG.

Moreover, the correlation between genotype of CYP1A2 and ZHENG types in FZHY efficacy was further analyzed in HBC. As shown in Table 7, there also was significant relevance (*P* < 0.05) between GA plus AA genotype and the efficacy index of deficiency ZHENG at CYP1A2-G2964A sites. 

## 4. Discussion

ZHENG, a profile of symptoms and signs as a series of clinical phenotypes, plays an important role in understanding the human homeostasis and guiding the applications of TCM treatment. All diagnostic and therapeutic methods in TCM are based on the differentiation of ZHENG, and this concept has been used for thousands of years in China [20]. The “Heat,” “Cold,” “Excess,” and “Deficiency” are the four basic syndromes in TCM. In TCM practice, an experiential diagnosis and efficacy evaluation approach has been frequently used to classify Excess, Deficiency, and Deficiency-Excess syndromes in HBC patients [21]. In order to demonstrate whether ZHENG differentiation approach leads to a personalized treatment, we investigated FZHY efficacy on HBC through a pharmacogenetic evaluation.

CYP1A2 is an important member of the cytochrome P450 superfamily [12], which is cytochrome P450 family 1, subfamily A, polypeptide 2. Its protein content contributes with 13% of the total CYP protein in liver [22]. CYP1A2 activity can be used to monitor the alteration of liver function in clinical practice [23]. Especially, CYP1A2 is involved in the metabolism of many drugs, environmental toxins, and endogenous substrates [24]. In contrast to exogenous factors such as smoking causing enzyme induction, to drug intake, and to dietary factors, the genetic influences on CYP1A2 enzyme activity has been shown [25]. It has been reported that CYP1A2 genotype is involved in the rate-limiting step in the metabolism of many drugs such as caffeine in myocardial infarction [26], theophylline in asthma [27], and clozapine in schizophrenia [28], as well as in the bioactivation of procarcinogens [29]. Besides, CYP1A2 phenotype is applied frequently in epidemiologic and drug-drug interaction studies [23].

Previous studies have shown that Chinese herbal formula, Erxian Soup, is associated with CYP1A2 genotype [13] and suggested that the male climacteric syndrome patients with CYP1A2-G2964A locus at GG genotype have higher efficiency and those with GA genotype have lower efficiency when treated with Erxian Soup. In this study, our results showed that ZHENG score are significantly decreased (*P* < 0.001) by FZHY treatment and suggested that FZHY improves the life quality of HBC patients. Moreover, there was significant correlation between ZHENG classification and FZHY efficacy (*P* < 0.05). In ZHENG efficacy of FZHY between effective and invalid groups, there was significant difference in CYP1A2-G2964A locus with AA compared to GG genotype (*P* < 0.05), and there also was significant relevance between GA plus AA genotype and GG in deficiency ZHENG (*P* < 0.05). It was suggested that HBC patients with CYP1A2-G2964A locus at GG genotype might have the higher efficiency and those with AA genotype have lower efficiency when treated with FZHY. However, there were no statistically significant between FZHY efficacy and Child-Pugh score in the comparison of any genetic locus. The study provided a proof for the ZHENG efficacy evaluation of Chinese herbal formula, which would be helpful to the clinical application of FZHY personalized treatment in HBC patients.

Since Chinese herbal formula contains multiple compounds, its pharmacological effects are the overall performance of these compounds interaction. Though our results showed that CYP1A2 genotypes might respond to the comprehensive effects of FZHY with multiple compounds based on ZHENG phenotype in HBC patients, it is still difficult to understand which compounds of FZHY respond to CYP1A2 genotype. Further researches might demonstrate the correlation between the interactions of FZHY contented compounds and the genotype of CYP and/or other drug metabolic genes through genome-wide association studies in a large number of HBC patients.

## 5. Conclusion

In this study, the FZHY efficacy between before and after treatment for 6 months was evaluated as a clinical outcome assessment. FZHY therapy improved clinical symptoms and ZHENG score in HBC. The efficacy may be related to CYP SNPs at CYP1A2-G2964A loci, suggesting that there is a possibility of ZHENG-based FZHY efficacy on HBC predicted by CYP1A2 genetic polymorphisms.

## Figures and Tables

**Table 1:** Clinical data of HBC patients.

	Patients (%)
Gender	
Male (%)	79 (71.17)
Female (%)	32 (28.83)
Mean age (yr)	49.51 ± 10.05
Child-Pugh classification	
A	89 (80.18)
B	20 (18.02)
C	2 (1.80)
ZHENG classification	
Excess	69 (62.16)
Deficiency-excess	21 (18.92)
Deficiency	21 (18.92)

**Table 2:** The gene position, polymorphism, and primer sequences of CYP SNPs.

Gene position	Rs number	Polymorphism	Primer sequences
CYP1A2-733	rs762551	C/A	F: 5′-CTACTCCAGCCCCAGAAGTG-3 R: 5′-CTGATGCGTGTTCTGTGCTT-3′
CYP1A2-2964	rs2069514	G/A	F: 5′-AACACAACGGGACTTCTTGG-3 R: 5′-GGCATGACAATTGCTTGAAT-3′
CYP1A2-5347	rs2470890	T/C	F: 5′-ATCTACGGGCTGACCATGAA-3 R: 5′-CTTGGCCTCCTAAAATGCTG-3′

**Table 3:** Effects of FZHY on ALT, AST, Child-Pugh score, and ZHENG sore in HBC.

Parameters	FZHY treatment (M ± *Q*) (*n* = 111)		*Z*	P*
	Before	After		
ALT (U/L)	34.50 ± 31.75	33.00 ± 25.00	−1.764	0.078
AST (U/L)	43.00 ± 19.62	41.00 ± 26.25	−1.445	0.149
Child-Pugh (score)	5.00 ± 0.00	5.00 ± 0.00	−0.536	0.592
ZHENG (score)	212.5 ± 100.5	111.00 ± 15.25	−7.673	**0.001**

*Wilcoxon test.

The bold font emphasizes the result was statistically significant (*P* < 0.05)

**Table 4:** Correlation between ZHENG and FZHY efficacy on HBC.

ZHENG classification	Effective (%) *n* = 67	Invalid (%) *n* = 44	*P* ^#^	OR	95% CI
Excess	39 (58.21)	30 (68.18)	0.611	0.854	0.464, 1.570
Deficiency-Excess	10 (14.93)	11 (25.00)	0.277	0.597	0.234, 1.524
Deficiency	18 (26.86)	3 (6.82)	**0.044**	3.940	1.095, 14.173

**Table 5:** Correlation between CYP1A2 genotypes and FZHY efficacy on Child-Pugh scores in HBC.

Genotypes	Effective (%) *n* = 25	Invalid (%) *n* = 86	*P* ^#^	OR	95% CI
CYP1A2-5347					
CC	17 (70.80)	63 (75.00)			
CT	6 (25.00)	20 (23.80)	0.844	0.899	0.312, 2.591
TT	1 (4.20)	1 (1.20)	0.393	0.270	0.016, 4.541
CT + TT	7 (29.20)	21 (25.00)	0.681	0.810	0.295, 2.221
CYP1A2-2964					
GG	10 (41.70)	46 (53.50)			
GA	11 (45.80)	30 (34.90)	0.289	0.593	0.224, 1.567
AA	3 (12.50)	10 (11.60)	0.700	0.725	0.168, 3.121
GA + AA	14 (58.30)	40 (46.50)	0.306	0.621	0.249, 1.552
CYP1A2-733					
CC	3 (12.50)	9 (10.50)			
CA	9 (37.50)	38 (44.20)	0.653	1.407	0.316, 6.277
AA	12 (50.00)	39 (45.30)	0.914	1.083	0.252, 4.656
CA + AA	21 (87.50)	77 (89.50)	0.777	1.222	0.304, 4.921

The bold font emphasizes the result was statistically significant (*P* < 0.05)

**Table 6:** Correlation between CYP1A2 Genotypes and FZHY efficacy on ZHENG score in HBC.

Genotypes	Effective (%) *n* = 67	Invalid (%) *n* = 44	*P* ^#^	OR	95% CI
CYP1A2-5347					
CC	48 (71.60)	32 (72.70)			
CT	17 (25.40)	9 (20.50)	0.624	0.794	0.315, 2.000
TT	0 (0)	2 (4.50)			
CT + TT	17 (25.40)	11 (25.00)	0.947	0.971	0.402, 2.341
CYP1A2-2964					
GG	33 (49.30)	23 (52.30)			
GA	30 (44.80)	11 (25.00)	0.146	0.526	0.220, 1.258
AA	3 (4.50)	10 (22.70)	**0.030**	4.783	1.184, 19.321
GA + AA	33 (49.30)	21 (47.70)	0.815	0.913	0.426, 1.959
CYP1A2-733			0.527		
CC	9 (13.40)	3 (6.80)			
CA	27 (40.30)	20 (45.50)	0.266	2.222	0.532, 9.275
AA	30 (44.80)	21 (47.70)	0.299	2.100	0.507, 8.694
CA + AA	57 (85.10)	41 (93.20)	0.261	2.158	0.550, 8.466

The bold font emphasizes the result was statistically significant (*P* < 0.05)

**Table 7:** Correlation between CYP1A2 genotype and ZHENG types in FZHY efficacy.

Genotypes	Excess ZHENG		*P* ^#^	Deficiency-Excess ZHENG		*P* ^#^	Deficiency ZHENG		*P* ^#^
	Effective (%) *n* = 39	Invalid (%) *n* = 30		Effective (%) *n* = 10	Invalid (%) *n* = 11		Effective (%) *n* = 18	Invalid (%) *n* = 3	
CYP1A2-5347									
CC	27 (69.23)	20 (66.67)		8 (80.00)	8 (72.73)		14 (77.78)	2 (66.67)	
CT	11 (28.21)	5 (16.67)	0.425	2 (20.00)	3 (27.27)	1.000	4 (22.22)	1 (33.33)	1.000
TT	0 (0)	2 (6.67)	—	0 (0)	0 (0)	—	0 (0)	0 (0)	—
CT + TT	11 (28.21)	7 (23.33)	0.788	2 (20.00)	3 (27.27)	1.000	4 (22.22)	1 (33.33)	1.000
CYP1A2-2964									
GG	17 (43.59)	15 (50.00)		7 (70.00)	5 (45.45)		1 (5.56)	2 (66.67)	
GA	19 (48.72)	7 (23.33)	0.119	3 (30.00)	3 (27.27)	1.000	12 (66.67)	1 (33.33)	0.071
AA	3 (7.69)	6 (20.00)	0.454	0 (0)	3 (27.27)	—	5 (27.78)	0 (0)	—
GA + AA	22 (56.41)	13 (43.33)	0.420	3 (30.00)	6 (54.55)	0.387	17 (94.44)	1 (33.33)	**0.041**
CYP1A2-733									
CC	5 (12.82)	2 (6.67)		2 (20.00)	1 (9.09)		2 (11.11)	0 (0)	
CA	14 (35.89)	13 (43.33)	0.426	6 (60.00)	4 (36.36)	1.000	8 (44.44)	2 (66.67)	—
AA	20 (51.28)	13 (43.33)	0.691	2 (20.00)	6 (54.55)	0.491	8 (44.44)	1 (33.33)	—
CA + AA	34 (87.18)	26 (86.67)	0.454	8 (80.00)	10 (90.91)	0.586	16 (88.88)	3 (100.00)	—

The bold font emphasizes the result was statistically significant (*P* < 0.05)

## References

[1] McMahon BJ (2005). Epidemiology and natural history of hepatitis B. *Seminars in Liver Disease*.

[2] Margolis HS, Alter MJ, Hadler SC (1991). Hepatitis B: evolving epidemiology and implications for control. *Seminars in Liver Disease*.

[3] De Jongh FE, Janssen HLA, De Man RA, Hop WCJ, Schalm SW, Van Blankenstein M (1992). Survival and prognostic indicators in hepatitis B surface antigen-positive cirrhosis of the liver. *Gastroenterology*.

[4] Li QY, Guo ZZ, Liang J (2012). Interleukin-10 genotype correlated to TCM syndrome in hepatitis B-caused cirrhosis. *Evidence-Based Complementary and Alternative Medicine*.

[5] Hu Y-Y, Liu P, Liu C (2006). Investigation on indication of fuzheng huayu capsule against hepatic fibrosis and its non-invasive efficacy evaluation parameters: data analysis of liver biopsy of 50 patients with chronic hepatitis B before and after treatment. *Zhongguo Zhong Xi Yi Jie He Za Zhi*.

[6] Liu P, Liu C, Xu L-M (1998). Effects of Fuzheng Huayu 319 recipe on liver fibrosis in chronic hepatitis B. *World Journal of Gastroenterology*.

[7] Liu P, Hu Y-Y, Liu C (2003). Multicenter clinical study about the action of Fuzheng Huayu Capsule against liver fibrosis with chronic hepatitis B. *Zhong Xi Yi Jie He Xue Bao*.

[8] Jiang CM, Liu CH, Liu C (2003). Inhibiting effect of Fuzhenghuayu Capsule on the activation of hepatic stellate cells in rats. *Chinese Journal of Integrated Traditional and Western Medicine*.

[9] Song YN, Sun JJ, Lu YY (2013). Therapeutic effect of Fuzheng-Huayu tablet based traditional Chinese medicine syndrome differentiation on hepatitis B caused cirrhosis. *Evidence-Based Complementary and Alternative Medicine*.

[10] Nebert DW, Russell DW (2002). Clinical importance of the cytochromes P450. *The Lancet*.

[11] Chen Q, Zhang T, Wang J-F, Wei D-Q (2011). Advances in human cytochrome P450 and personalized medicine. *Current Drug Metabolism*.

[12] Nelson DR, Koymans L, Kamataki T (1996). P450 superfamily: update on new sequences, gene mapping, accession numbers and nomenclature. *Pharmacogenetics*.

[13] Yang M, Ding CY, Zhang X (2008). Genetic association research between mutation in CYP1A2 gene and erxian soup response. *Chinese Journal of Information on TCM*.

[14] Chinese Association of Integrative Medicine (2006). Guideline for the diagnosis and treatment of liver fibrosis with integrative. Medicine. *Zhong Xi Yi Jie He Xue Bao*.

[15] Wang Q-L, Yuan J-L, Tao Y-Y, Zhang Y, Liu P, Liu C-H (2010). Fuzheng Huayu recipe and vitamin E reverse renal interstitial fibrosis through counteracting TGF-*β*1-induced epithelial-to-mesenchymal transition. *Journal of Ethnopharmacology*.

[16] Cholongitas E, Papatheodoridis GV, Vangeli M, Terreni N, Patch D, Burroughs AK (2005). Systematic review: the model for end-stage liver disease—should it replace Child-Pugh’s classification for assessing prognosis in cirrhosis?. *Alimentary Pharmacology and Therapeutics*.

[17] Christensen E (2004). Prognostic models including the Child-Pugh, MELD and Mayo risk scores—where are we and where should we go?. *Journal of Hepatology*.

[18] Zheng X (2002). *Guideline for Clinical New Drug Research in Chinese Herbal Medicine*.

[19] Filippini S, Blanco A, Fernández-Marmiesse A (2007). Multiplex SNaPshot for detection of BRCA1/2 common mutations in Spanish and Spanish related breast/ovarian cancer families. *BMC Medical Genetics*.

[20] Wang B-E (2000). Treatment of chronic liver diseases with traditional Chinese medicine. *Journal of Gastroenterology and Hepatology*.

[21] Song YN, Zhang H, Guan Y (2012). Classification of traditional Chinese medicine syndromes in patient with chronic hepatitis B by SELDI-based protein-chip analysis. *Evidence-Based Complementary and Alternative Medicine*.

[22] Shimada T, Yamazaki H, Mimura M, Inui Y, Guengerich FP (1994). Interindividual variations in human liver cytochrome P-450 enzymes involved in the oxidation of drugs, carcinogens and toxic chemicals: studies with liver microsomes of 30 Japanese and 30 Caucasians. *Journal of Pharmacology and Experimental Therapeutics*.

[23] Faber MS, Jetter A, Fuhr U (2005). Assessment of CYP1A2 activity in clinical practice: why, how, and when?. *Basic and Clinical Pharmacology and Toxicology*.

[24] Shou M, Korzekwa KR, Brooks EN, Krausz KW, Gonzalez FJ, Gelboin HV (1997). Role of human hepatic cytochrome P450 1A2 and 3A4 in the metabolic activation of estrone. *Carcinogenesis*.

[25] Rasmussen BB, Brix TH, Kyvik KO, Brøsen K (2002). The interindividual differences in the 3-demthylation of caffeine alias CYP1A2 is determined by both genetic and environmental factors. *Pharmacogenetics*.

[26] Cornelis MC, El-Sohemy A, Kabagambe EK, Campos H (2006). Coffee, CYP1A2 genotype, and risk of myocardial infarction. *Journal of the American Medical Association*.

[27] Obase Y, Shimoda T, Kawano T (2003). Polymorphisms in the CTP1A2 gene and theophylline metabolism in patients with asthma. *Clinical Pharmacology and Therapeutics*.

[28] Balibey H, Basoglu C, Lundgren S (2011). CYP1A21F polymorphism decreases clinical response to clozapine in patients with schizophrenia. *Bulletin of Clinical Psychopharmacology*.

[29] Eaton DL, Gallagher EP, Bammler TK, Kunze KL (1995). Role of cytochrome P4501A2 in chemical carcinogenesis: implications for human variability in expression and enzyme activity. *Pharmacogenetics*.

